# Clinical outcomes of esophageal granular cell tumors with different endoscopic resection methods

**DOI:** 10.1038/s41598-023-37998-x

**Published:** 2023-07-03

**Authors:** Dae Gon Ryu, Cheol Woong Choi, Su Jin Kim, Chung Su Hwang, Dae Hwan Kang, Hyung Wook Kim, Su Bum Park, Bong Soo Son

**Affiliations:** 1 Department of Internal Medicine, Medical Research Institute, Pusan National University School of Medicine and Research Institute for Convergence of Biomedical Science and Technology, Pusan National University Yangsan Hospital, Beomeo-ri Mulgeum-eup, Yangsan-si, 50612 Gyeongsangnam-do Korea; 2 Department of Pathology, Pusan National University Yangsan Hospital, Yangsan, Korea; 3 Department of Thoracic and Cardiovascular Surgery, Pusan National University School of Medicine and Research Institute for Convergence of Biomedical Science and Technology, Pusan National University Yangsan Hospital, Yangsan, Korea

**Keywords:** Gastroenterology, Signs and symptoms

## Abstract

Esophageal granular cell tumors (GCTs), the second most common subepithelial tumors (SETs) of the esophagus, are potentially malignant with no definite management guidelines available. We retrospectively enrolled 35 patients with endoscopically resected esophageal GCTs between December 2008 and October 2021 and evaluated the clinical outcomes from the various methods performed. Several modified endoscopic mucosal resections (EMRs) were performed for treating esophageal GCTs. Clinical and endoscopic outcomes were evaluated. Mean age of patients was 55.8 ± 8.2, with majority being men (57.1%). Mean tumor size was 7.2 ± 2.6 mm, most (80.0%) were asymptomatic and present in the distal third of the esophagus (77.1%). Endoscopic characteristics predominantly included broad-based (85.7%) and whitish-to-yellowish color changes (97.1%). Endoscopic ultrasound (EUS) of 82.9% of the tumors revealed homogeneous hypoechoic SETs originating from the submucosa. The five endoscopic treatment methods used were: ligation-assisted (77.1%), conventional (8.7%), cap-assisted (5.7%), and underwater (5.7%) EMRs and ESD (2.9%). Mean procedure time was 6.6 ± 2.1 min, and no procedure-associated complications were noted. The en-bloc and complete histologic resection rates were 100% and 94.3%, respectively. No recurrences were noted during follow-up, and no significant differences in the clinical outcomes of the different methods of endoscopic resection were found. Based on tumor characteristics and therapeutic outcomes, modified EMR methods can be effective and safe. However, there were no significant differences in the clinical outcomes of the different methods of endoscopic resection.

## Introduction

Esophageal granular cell tumors (GCTs) are rare soft tissue tumors derived from Schwann cells and are potentially malignant^1,2^. The most common esophageal subepithelial tumors (SETs) are leiomyomas, followed by esophageal GCTs^3,4^. Resection for incidental, benign esophageal SETs, such as leiomyomas, is usually needless, unless symptomatic or complicated. Although most GCTs are indolent or benign slow-progressing tumors, a few are malignant (less than 2%)^2^. Therefore, despite usually finding GCTs incidentally and without symptoms or complications, curative resection of esophageal GCTs should be considered. Consequently, it is necessary to determine a safe resection method for treating esophageal GCTs.

Management plans could be more easily determined if obtaining a definite diagnosis of esophageal SETs was possible. However, this is difficult without tissue acquisition as SETs, including GCTs, are located below the epithelial layers. Additionally, since the tumors are located below the normal epithelium, the diagnostic yield may be insufficient to diagnose esophageal SETs via endoscopic forceps biopsy^5^. While endoscopic ultrasound (EUS) is useful for characterizing the echogenicity, exact tumor size, and layer of origin of SETs, accurate diagnostic rates without tissue acquisition are between 45.5 and 66.3%^6,7^. Therefore, diagnostic endoscopic resection of tumors within the submucosa and without invasion into the proper muscle layer may result in a definite diagnosis of esophageal SETs and, consequently, curative endoscopic resection of the tumors.

As esophageal GCTs are rare, there is currently no recommended method of endoscopic resection. Although various methods of endoscopic resection, such as conventional endoscopic mucosal resection (EMR), modified EMR using a cap or band-ligation device, and endoscopic submucosal dissection (ESD), have been reported for esophageal GCTs located within the submucosa^8–11^, all were small case series.

Therefore, the aim of this relatively larger series of pathologically confirmed esophageal GCTs was to evaluate treatment outcomes of different methods of endoscopic resection.

## Patients and methods

### Patients

Thirty-five patients with endoscopically resected esophageal GCTs were retrospectively selected from the patient database at Pusan National University Yangsan Hospital between December 2008 and October 2021. The present study was approved by the Ethics Committee of Pusan National University Yangsan Hospital, where this study was performed (institutional review board no. 05-2022-033). Informed consent was waived by the ethics committee (Institutional Review Board of Pusan National University Yangsan Hospital) because the subject’s medical records were anonymized before analysis. The study was conducted in accordance with the principles of the Declaration of Helsinki.

### Histopathology

The resected or biopsied specimens were fixed in 10% formalin, embedded in paraffin wax, and sliced into 2-mm-thickness sections. The tissue sections were stained with hematoxylin and eosin, anti-S-100 antibody, anti-smooth muscle actin antibody, and c-kit (CD117). GCTs were diagnosed as S-100-positive tumors^12,13^. All tissue slides were reviewed in a blinded manner by two pathologists, and discordant cases were re-evaluated under a multi-headed microscope to reach consensus. En bloc resections involved resecting intact tumors in one piece, and complete histologic resections were defined by the absence of tumor cells at the resected margins of en bloc resected tumors.

### Conventional endoscopic and endoscopic ultrasound examinations

Following the detection of esophageal SETs, several endoscopic findings were recorded. All the enrolled esophageal SETs were firm. Lesion size was determined from pathologic specimens and lesion location was classified relative to their distance from the incisor teeth: upper third (15–24 cm), middle third (24–32 cm), and lower third (32–40 cm) of the esophagus. Erosive esophagitis was defined by definite esophageal mucosal erosion. Gross type tumors were classified as either narrow-necked or broad-based. Narrow-necked tumors indicate those with elevated lesions with a clear notched base or peduncle, while broad-based tumors have elevated lesions without a notch or peduncle^14^. The color of the overlying mucosa was recorded as white-to-yellowish or reddish compared to the surrounding normal esophageal mucosa. The mucosal surface was classified as having a round, flat, or cobblestone/molar tooth appearance.

EUS was performed on 30 GCTs and 11 leiomyomas using a high-frequency (20 MHz) catheter probe (UM3D-DP20-25R, Olympus Co. Ltd., Tokyo, Japan) and the water-filled method. All examinations were performed under intravenous conscious sedation with midazolam (2.5–8 mg). The images of the approximated 5–10 endosonograms performed on each patient, were reviewed by three experienced endosonographers (CW Choi, SJ Kim, and DG Ryu), blinded to the final diagnosis, who had previously performed more than 1000 examinations. Discordant cases were re-evaluated to obtain consensus. The following endoscopic and EUS features were recorded for all the tumors: (1) maximal diameter; (2) echogenicity in comparison with the normal proper muscle layer (hyper- or hypoechoic); and (3) homogeneity (homogenous or heterogeneous).

### Endoscopic resection

This study used five different endoscopic resection techniques: four types of EMR (conventional, ligation-assisted, cap-assisted, and underwater) and ESD. All endoscopic resections were performed under intravenous conscious sedation with midazolam (2.5–8 mg). Patients were started on a soft diet the day after successful endoscopic resection. Operation time was calculated from the first photograph of the tumor to the last, which was taken after endoscopic resection (just after the hemostatic procedure of the artificial ulcer bed).

#### Conventional EMR

**Figure 1:**
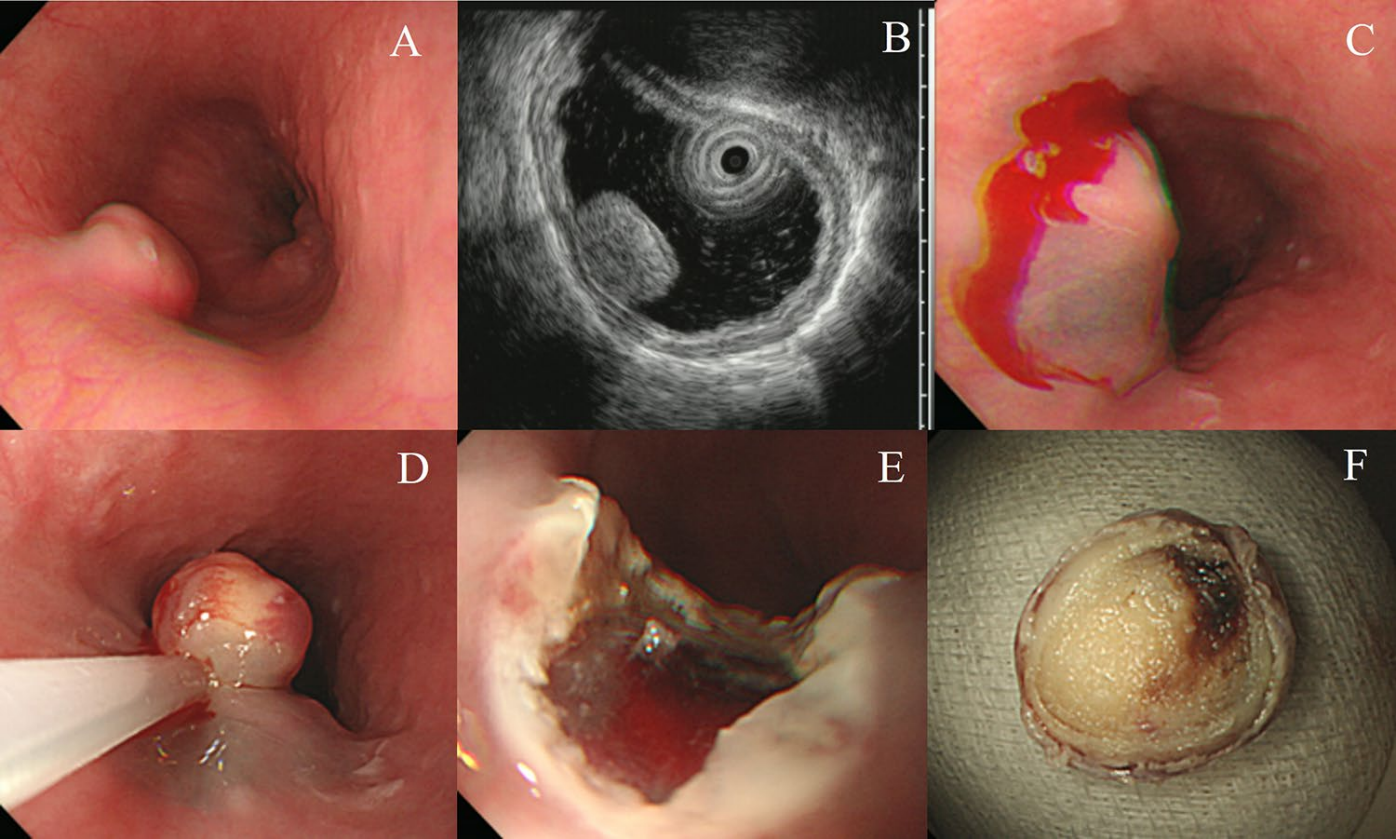
Conventional endoscopic mucosal resection for a 12-mm-sized esophageal granular cell tumor. A narrow-necked, round subepithelial tumor (SET) is detected in the lower esophagus (**A**). Endoscopic ultrasound (EUS) revealed a hypoechoic mass without invasion into the proper muscle layer (**B**). After submucosal injection (**C**), electrosurgical snaring is performed (**D**). The ulcer bed after endoscopic resection (**E**). An en-bloc resection was achieved (**F**).

All procedures were performed using a single-channel endoscope (H260 or H290; Olympus Optical Co. Ltd., Tokyo, Japan) with a transparent cap attached. After injecting normal saline with a mixture of epinephrine and indigo carmine into the submucosa, endoscopic maneuvers were selected based on the endoscopists’ decision and tumor morphology. Conventional EMR was performed after the submucosa injection using an endoscopic electrosurgical snare and electrosurgical generator (Endocut Q current, effect 3, cut duration 2, cut interval 5, VIO300D electrosurgical unit, ERBE, Tübingen, Germany) (Fig. 1).

#### Ligation-assisted EMR

**Figure 2:**
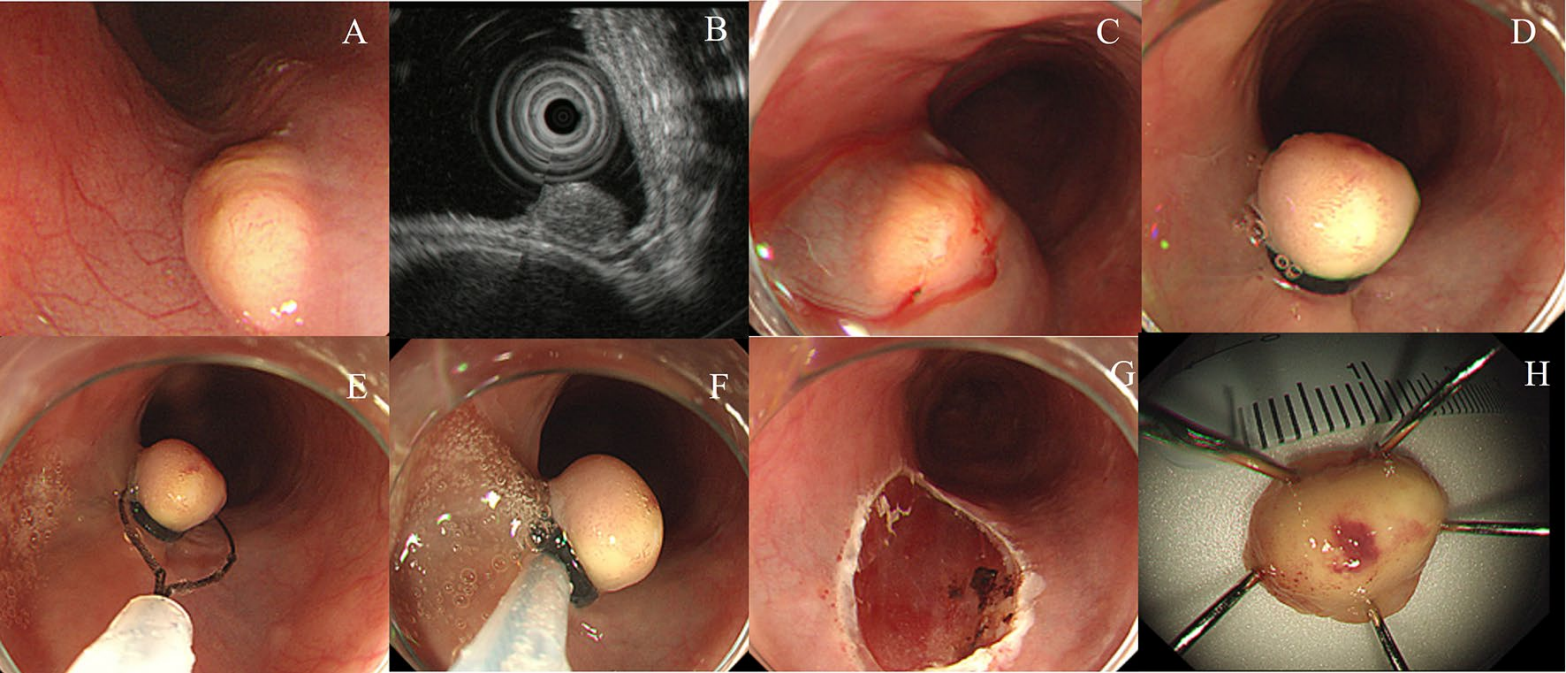
Ligation-assisted endoscopic mucosal resection for a 10-mm-sized esophageal granular cell tumor. A broad-based, round subepithelial tumor (SET) with whitish-to-yellowish overlying mucosa is detected in the middle esophagus (**A**). Endoscopic ultrasound (EUS) revealed a hypoechoic mass originating from the submucosa (**B**). After submucosal injection (**C**), band-ligation is performed (**D**). Electrosurgical snaring is performed under the band (**E**, **F**). The ulcer bed after endoscopic resection (**G**). An en-bloc resection was achieved (**H**).

All procedures were performed using a single-channel endoscope (H260 or H290; Olympus Optical Co. Ltd., Tokyo, Japan) with a transparent cap attached. After injecting normal saline with a mixture of epinephrine and indigo carmine into the submucosa, we inserted an endoscope with a band ligation device attached to its tip (Stiegmann-Goff ClearVue; ConMed, Boston, MA). After the tumor had been aspirated into the cap of the ligation device, the elastic band was deployed beneath the main tumor. Subsequently, we performed endoscopic resection beneath the elastic band using the same endoscopic electrosurgical snare and electrosurgical generator (Endocut Q current, effect 3, cut duration 2, cut interval 5, VIO300D electrosurgical unit, ERBE, Tübingen , Germany) (Fig. 2).

#### Cap-assisted EMR

**Figure 3:**
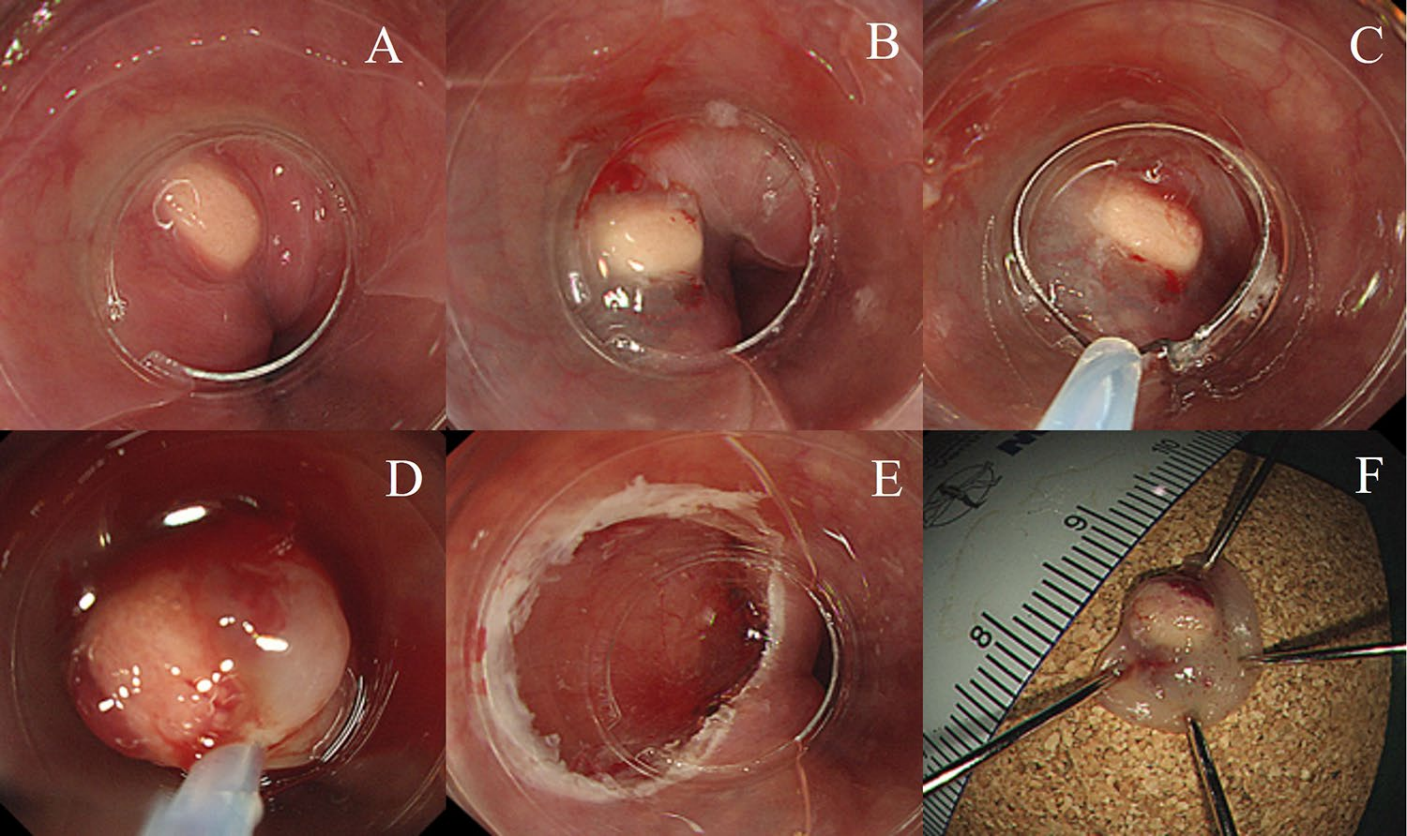
Cap-assisted endoscopic mucosal resection for a 5-mm-sized esophageal granular cell tumor. A broad-based, round subepithelial tumor (SET) with whitish-to-yellowish overlying mucosa is seen in the lower esophagus (**A**). After submucosal injection (**B**), the crescent-shaped snare is positioned on the internal circumferential ridge at the tip of the oblique cap (**C**). Electrosurgical snaring is performed under the band (**D**). The ulcer bed after endoscopic resection (**E**). An en-bloc resection was achieved (**F**).

We used a single-channel endoscope (H260 or H290; Olympus Optical Co. Ltd., Tokyo, Japan) with an oblique distal cap attachment (MAJ-290, Olympus Medical Systems Corp.) and a 25-mm single-use crescent electrosurgical snare (SD-221L-25, Olympus Medical Systems Corp.). After submucosal injection, the crescent-shaped snare was positioned on the internal circumferential ridge at the tip of the oblique cap. The lesion was pulled into the cap using the suction function and then snared. Endoscopic resection was performed using the same electrosurgical unit described above (VIO300D, ERBE, Tübingen, Germany) (Fig. 3).

#### Underwater EMR

**Figure 4:**
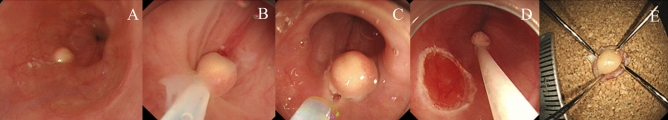
Underwater endoscopic mucosal resection for a 6-mm-sized esophageal granular cell tumor. A broad-based, round subepithelial tumor (SET) with whitish-to-yellowish overlying mucosa is seen in the lower esophagus (**A**). After filling the lumen of the esophagus with distilled water (**B**), electrosurgical snaring is performed without submucosal injection (**C**). The ulcer bed after endoscopic resection (**D**). An en-bloc resection was achieved (**E**).

We used a two-channel endoscope (GIF-2TQ260M; Olympus, Tokyo, Japan) and performed underwater EMR in the lateral decubitus position with a 30° head-up tilt to prevent aspiration pneumonia. One channel was used to infuse water, and the other to introduce an electrosurgical snare. After filling the lumen of the esophagus with distilled water, electrosurgical snaring was performed without submucosal fluid injection (Fig. 4).

##### ESD

**Figure 5:**
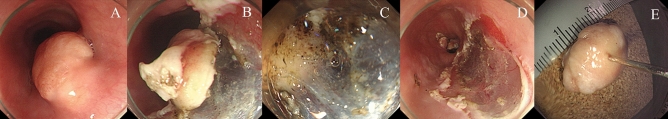
Endoscopic submucosal dissection for a 17-mm-sized esophageal granular cell tumor. A broad-based, round subepithelial tumor (SET) with whitish-to-yellowish overlying mucosa is seen in the upper esophagus (**A**). After submucosal injection, submucosal dissection was performed (**B**, **C**). The ulcer bed after endoscopic resection (**D**). An en-bloc resection was achieved (**E**).

This was performed using a single-channel endoscope (H260 or H290; Olympus Optical Co. Ltd., Tokyo, Japan) and the electrosurgical unit (VIO300D, ERBE, Tübingen, Germany). After lifting the tumor with submucosal injection, a circumferential incision was made around the lesion, and submucosal dissection was performed using the DualKnife™ electrosurgical knife (Olympus Medical Systems Corp.) (Fig. 5).

### Statistical analysis

Statistical analyses were performed for each lesion. Associations between variables of the different groups were assessed using the chi-square test, and patient age and tumor size were assessed using the Student’s t-test. Statistical significance was set at *p* < 0.05. The data were analyzed using PASW Statistics for Windows, Version 21.0 (SPSS Inc., Chicago, IL).

### Ethical standard

The study was approved by the ethics committee of the Institutional Review Board of Pusan National University Yangsan Hospital (Institutional Review Board no. 05–2022-033). There were no conflicts of interest or sponsors in this study.

## Results

### Baseline characteristics

During the study period, 35 esophageal GCTs were removed using endoscopic techniques. The mean patient age was 55.8 ± 8.2 years, and 57.1% were male. The most common symptom was reflux (11.4%), and most tumors were detected during the endoscopic screening (80.0%). The mean follow-up duration was 54.6 ± 72.9 months (Table 1).

**Table 1:** Baseline characteristics of the enrolled patients with esophageal granular cell tumors.

Characteristics	Conventional EMR (n = 3)	Ligation-assisted EMR (n = 27)	Cap-assisted EMR (n = 2)	Underwater-EMR (n = 2)	ESD (n = 1)	Total (n = 35)
Male, Sex, n (%)	1 (33.3)	15 (55.6)	2 (100)	1 (50)	1 (100)	20 (57.1)
Age, years,						
mean ± SD	55.0 (2.6)	55.1 (8.0)	56.5 (8.5)	69.0 (1.4)	49	55.8 (8.2)
Symptoms, n (%)						
Without symptoms	1 (33.3)	23 (85.2)	1 (50)	2 (100)	1 (100)	28 (80.0)
Reflux	1 (33.3)	2 (5.7)	1 (50)	0 (0)	0 (0)	4 (11.4)
Dyspepsia	0 (0)	1 (3.7)	0 (0)	0 (0)	0 (0)	1 (2.9)
Epigastric pain	0 (0)	1 (3.7)	0 (0)	0 (0)	0 (0)	1 (2.9)
Globus	1 (33.3)	0 (0)	0 (0)	0 (0)	0 (0)	1 (2.9)
Follow up months, mean (SD)	51.9 (3.0)	56.2 (74.6)	113.4 (138.2)	26.5 (6.2)	26.1	54.6 (72.9)

EMR, endoscopic mucosal resection; ESD, endoscopic submucosal dissection; N, number; SD, standard deviation.

### Endoscopic features

Nineteen GCTs (54.3%, 19/35) were pathologically confirmed by endoscopic forceps biopsy before endoscopic resection. The mean tumor size was 7.2 ± 2.6 mm, and the main location was the lower third of the esophagus (77.1%). Only 14.3% of the tumors were associated with erosive esophagitis. Broad-based morphology (85.7%) and whitish-to-yellowish color changes (97.1%) were the most common endoscopic features of the esophageal GCTs (Table 2).

**Table 2:** Endoscopic features of the enrolled patients with esophageal granular cell tumors.

Characteristics	Conventional EMR (n = 3)	Ligation-assisted EMR (n = 27)	Cap-assisted EMR (n = 2)	Underwater-EMR (n = 2)	ESD (n = 1)	Total (n = 35)
Pre-EMR biopsy, n(%)						
Granular cell tumor	1 (33.3)	16 (59.3)	1 (50)	0 (0)	1 (100)	19 (54.3)
Acanthosis	1 (33.3)	5 (18.5)	0 (0)	0 (0)	0 (0)	6 (17.1)
Hyperplastic squamous epithelium	0 (0)	1 (3.7)	0 (0)	0 (0)	0 (0)	1 (2.9)
Squamous epithelium	0 (0)	0 (0)	0 (0)	1 (50)	0 (0)	1 (2.9)
Lesion size (mm, mean ± SD)	6.3 (4.9)	7.1 (2.1)	4.5 (0.7)	9.0 (4.2)	12	7.2 (2.6)
Tumor location, longitudinal, n (%)						
Lower third	2 (66.7)	22 (81.5)	1 (50)	2 (100)	0 (0)	27 (77.1)
Middle third	0 (0)	1 (3.7)	0 (0)	0 (0)	0 (0)	1 (2.9)
Upper third	1 (33.3)	4 (14.8)	1 (50)	0 (0)	1 (100)	7 (20.0)
Erosive esophagitis, n (%)	0 (0)	4 (80.0)	1 (50)	0 (0)	0 (0)	5 (14.3)
Gross type, n (%)						
Narrow neck	1 (33.3)	4 (14.8)	0 (0)	0 (0)	0 (0)	5 (14.3)
Broad base	2 (66.7)	23 (85.2)	2 (100)	2 (100)	1 (100)	30 (85.7)
Surface appearance, n (%)						
Flat	0 (0)	8 (29.6)	2 (100)	0 (0)	0 (0)	10 (28.6)
Cobble stone	1 (33.3)	9 (33.3)	0 (0)	2 (100)	0 (0)	12 (34.3)
Round	2 (66.7)	10 (37.0)	0 (0)	0 (0)	1 (100)	13 (37.1)
Surface Color, n (%)						
Normal	0 (0)	1 (3.7)	0 (0)	0 (0)	0 (0)	1 (2.9)
Whitish to yellowish	3 (100)	26 (96.3)	2 (100)	2 (100)	1 (100)	34 (97.1)
EUS, n (%)	2 (66.7)	25 (92.6)	0 (100)	1 (50)	1 (100)	29 (82.9)
EUS echo, n (%)						
Hyperechoic than proper muscle	2 (66.7)	22 (81.5)	0 (0)	1 (50)	0 (0)	25 (71.4)
Hypoechoic than proper muscle	0 (0)	3 (11.1)	0 (0)	0 (0)	1 (100)	4 (11.4)
Unchecked	1 (33.3)	2 (5.7)	2 (100)	1 (50)	0 (0)	6 (17.1)

EMR, endoscopic mucosal resection; ESD, endoscopic submucosal dissection; N, number; SD, standard deviation; EUS, endoscopic ultrasound.

EUS examinations performed on 29 of the 35 patients (82.9%) revealed homogeneous hypoechoic SETs originating from the submucosal layers. However, most GCTs (71.4%, 25/35) showed greater echogenicity than the proper muscle layer; no evidence of tumor invasion into the proper muscle layer was noted (Table 2).

### Endoscopic resection outcomes

Five different techniques of endoscopic resection were used: ligation-assisted (77.1%), conventional (8.7%), cap-assisted (5.7%), and underwater (5.7%) EMRs and ESD (2.9%). The mean operation time was 6.6 ± 2.1 min. No procedure-associated complications, such as perforation and delayed bleeding, were noted. Approximately half (48.6%) the patients had no complaints postoperatively. En bloc resection was achieved in all the endoscopic resections. No patient had lymphovascular invasion. Histologically clear resection margins were evident in 94.3% of the patients. No additional treatment was provided to the two patients with tumors with undetermined (n = 1) or vertical margin (n = 1) involvement, and no evidence of local recurrence was noted during follow-up. No significant differences were evident in the clinical outcomes of the different methods of endoscopic resection (Table 3). The largest esophageal GCT removed by EMR was 12 mm in size with a narrow neck-based morphology (Fig. 1); for ESD, this was 17 mm in size (Fig. 5). All the other tumors were less than 10 mm in maximal diameter. There was no distant metastasis or recurrence during the follow-up period.

**Table 3:** Clinical outcomes of the different methods of endoscopic resection of esophageal granular cell tumors.

Characteristics	Conventional EMR (n = 3)	Ligation-assisted EMR (n = 27)	Cap-assisted EMR (n = 2)	Underwater-EMR (n = 2)	ESD (n = 1)	Total (n = 35)
Operating time (min, mean ± SD)	4.3 (2.0)	6.5 (1.9)	7 (0)	7.5 (2.1)	12	6.6 (2.1)
Symptoms after endoscopic resection, n (%)						
Epigastric pain	0 (0)	11 (40.7)	0 (0)	1 (50)	0 (0)	12 (34.3)
Chest pain	1 (33.3)	4 (14.8)	0 (0)	0 (0)	0 (0)	5 (14.3)
Headache	0 (0)	1 (3.7)	0 (0)	0 (0)	0 (0)	1 (2.9)
Nothing	2 (66.7)	11 (40.7)	2 (100)	1 (50)	1 (100)	17 (48.6)
Resection margin, n (%)						
Clear	2 (66.7)	26 (96.3)	2 (100)	2 (100)	1 (100)	33 (94.3)
Undetermined	1 (33.3)	0 (0)	0 (0)	0 (0)	0 (0)	1 (2.9)
Vertical margin involvement	0 (0)	1 (3.7)	0 (0)	0 (0)	0 (0)	1 (2.9)
Lymphovascular invasion, n (%)	0 (0)	0 (0)	0 (0)	0 (0)	0 (0)	0 (0)

EMR, endoscopic mucosal resection; ESD, endoscopic submucosal dissection; N, number; SD, standard deviation.

## Discussion

In the present study, all sorts of endoscopic resection methods were safe and effective for removing esophageal EGC confined in the submucosa. The management plan of esophageal GCT is not established. Because of its indolent course, follow-up examinations without resection might be a management option. However, malignant GCTs have been reported in rare cases (less than 2% of GCTs)^2^. Therefore, curative resection of esophageal GCTs should be considered if tumors are feasible for endoscopic resection. In general, tumors located in the submucosa without evidence of proper muscle layer are accepted as indication of endoscopic treatment methods. In the present study, we recommended EUS examination before endoscopic resection to determine the tumor location within the esophageal wall (82.9% of patients). Various useful endoscopic resection methods have been reported: conventional EMR, ligation-assisted EMR, cap-assisted EMR, and ESD^8–11^. In the present study, we used various methods to remove esophageal GCTs in the submucosa, and all modalities showed a 100% en-bloc resection rate. Although the complete pathologic resection rate for conventional EMR was lower (66.7%) than that of other methods (96.3–100%), the case number of conventional EMR was only 3. Therefore, statistical analysis was impossible to evaluate the differences among treatment modalities. In the present study, however, because all artificial ulcer bed showed no definite remnant tumors after endoscopic resection regardless of histologic clear resection margin, we did not perform additional endoscopic resection or destructive treatment like argon plasma coagulation for patients with incomplete resection margin. No evidence of local recurrence was found in all patients during follow-up periods. Except for ESD, the operation time was less than 10 min in all EMR methods. Though most patients had no complains after endoscopic resection, common symptoms after the procedure were epigastric pain (34.3%) and chest pain (14.3%) which were easily controlled by proton pump inhibitors and antacids. In addition, procedure related significant complications like perforation or delayed bleeding were absent. According to the present study, all reported endoscopic resection methods could be used for esophageal GCTs.

Endoscopic resection of esophageal GCT has more advantages than regular follow up without resection. First, although conventional endoscopic examination and EUS could be used to diagnose and evaluate the risk of malignant potential of SETs, definite diagnosis is impossible before adequate tissue acquisition. The diagnostic yield of additional EUS examinations was reported as 45.5–66.3% among SETs^6,7^. Therefore, tissue acquisition should be considered to determine management plan. Secondly, endoscopic forceps biopsy is a simple method to obtain tissue for epithelial tumors. However, diagnostic yield of endoscopic forceps biopsy for esophageal SET may be suboptimal because the main tumors are located beneath the epithelial layer. A bite-on-bite technique for esophageal epithelial tumors could yield up to 54% of diagnostic rate with a higher risk of hemorrhage requiring endoscopic hemostasis^5^. In the present study, the diagnostic yield of esophageal GCTs by endoscopic forceps biopsy was only 54.3%. In contrast to endoscopic forceps biopsy, endoscopic resection using modified EMR or ESD could resect tumors in one-piece. Therefore, definite pathologic diagnosis and curative resection could be achieved simultaneously. If the resected specimen shows benign tumors such as leiomyoma or benign GCT, no additional examinations are needless. Thirdly, compared with EUS-fine needle aspiration and biopsy method, the EMR procedure could be performed by most endoscopist without additional linear EUS scope. Fourth, the procedure time of EMR or the modified EMR technique was short (within 10 min).

In the present study, the mean lesion size of esophageal GCTs was 7.2 mm. Only two cases were larger than 10 mm (12 mm for conventional EMR and 17 mm for ESD). The simplest endoscopic resection method for esophageal tumor is conventional EMR using an endoscopic snare. However, because some SETs, such as those with broad-based morphology may sink or flatten after submucosa fluid injection, endoscopic snaring of esophageal GCTs may be difficult. Therefore, various types of modified EMR methods have been developed. In the present study, 77.1% of esophageal GCTs were removed by ligation-assisted EMR. Ligation-assisted EMR achieved 100% en-bloc resection and 96.3% complete resection rates. After elastic banding deployment, the endoscopic snare was placed under the ligation band before resection. Although the snare may be placed above the elastic band to decrease iatrogenic perforation, a clear resection margin may be difficult, especially a clear vertical margin. A similar modified EMR technique with ligation-assisted EMR is cap-assisted EMR. The advantage of cap-assisted EMR is that it is simpler than ligation-assisted EMR or ESD. During cap-assisted EMR, after submucosal fluid injection, the tumor is suck into the cap and the prepositioned snare can capture the tumor base. Another modified EMR method used in the present study was underwater EMR. Underwater EMR was first proposed for the colorectal neoplasm. After filling the lumen with water, the tumor is lifted and floats away from the muscularis propria without submucosal fluid injection^15^. A previous study has reported the safe removal of rectal neuroendocrine tumors via underwater EMR^15^. Recently, underwater EMR has been attempted for the resection of tumors in the upper gastrointestinal tract^16,17^. Here, two esophageal GCTs were successfully removed using underwater EMR. However, there is no evidence of underwater EMR for esophageal lesions, and the risk of aspiration is high. In addition, suction may be useful when snaring submucosal lesions, but suction is difficult if the esophagus is filled with water. Since electrosurgical snare resection is limited in tumors larger than 10 mm, we performed ESD for the esophageal GCT with a diameter of 17 mm. Generally, owing to the relatively higher perforation risk of ESD compared to the various EMR techniques, it should only be performed by experts in ESD surgery. Here, smaller esophageal GCTs, particularly those less than 10 mm in maximal diameter, were able to be safely and effectively resected using different methods of EMR. We recommend using a modified EMR technique that is available in an individual institution to achieve higher complete histologic resection rates.

One study limitation was possible selection bias when retrospectively reviewing medical records. Additionally, as this study was conducted at a single center with a small number of patients, the results cannot be generalized to all patients with GCTs. Furthermore, we could not statistically analyze the clinical outcomes of the different methods of endoscopic resection. Given the rarity of esophageal GCTs, the additive effect of combining these findings with those of previous studies may be useful for managing esophageal GCTs. Another limitation is that most of the lesions were less than 1 cm in size, which generally warrants follow-up rather than excision. However, GCT has malignant potential, and according to our results, lesions smaller than 1 cm could be safely removed with modified EMR. In addition, EUS is required before endoscopic resection even for small lesions, but some patients underwent resection without EUS due to cost issues or reluctance to undergo additional examination.

In conclusion, most esophageal GCTs located within the submucosa can be excised via endoscopic resection. For small esophageal GCTs, particularly those less than 10 mm in maximal diameter, we recommend using modified EMR methods, including ligation-assisted, cap-assisted, or underwater EMR, in accordance with the endoscopists’ preferences and available endoscopic equipment in the individual institution. ESD may be an option when experiencing difficulty snaring SETs, especially those larger than 10 mm in size.

## Data Availability

We uploaded the data of our study on the website: https://github.com/gon22gon/E_GCT_ER for the purpose of academic sharing.
